# Cancer Metabolism: Phenotype, Signaling and Therapeutic Targets

**DOI:** 10.3390/cells9102308

**Published:** 2020-10-16

**Authors:** Jae Hyung Park, Woo Yang Pyun, Hyun Woo Park

**Affiliations:** Department of Biochemistry, College of Life Science and Biotechnology, Yonsei University, Seoul 03722, Korea; jhpark.main@yonsei.ac.kr (J.H.P.); kpwy910@yonsei.ac.kr (W.Y.P.)

**Keywords:** cancer metabolism, cell signaling, drug development, metabolic plasticity

## Abstract

Aberrant metabolism is a major hallmark of cancer. Abnormal cancer metabolism, such as aerobic glycolysis and increased anabolic pathways, has important roles in tumorigenesis, metastasis, drug resistance, and cancer stem cells. Well-known oncogenic signaling pathways, such as phosphoinositide 3-kinase (PI3K)/AKT, Myc, and Hippo pathway, mediate metabolic gene expression and increase metabolic enzyme activities. Vice versa, deregulated metabolic pathways contribute to defects in cellular signal transduction pathways, which in turn provide energy, building blocks, and redox potentials for unrestrained cancer cell proliferation. Studies and clinical trials are being performed that focus on the inhibition of metabolic enzymes by small molecules or dietary interventions (e.g., fasting, calorie restriction, and intermittent fasting). Similar to genetic heterogeneity, the metabolic phenotypes of cancers are highly heterogeneous. This heterogeneity results from diverse cues in the tumor microenvironment and genetic mutations. Hence, overcoming metabolic plasticity is an important goal of modern cancer therapeutics. This review highlights recent findings on the metabolic phenotypes of cancer and elucidates the interactions between signal transduction pathways and metabolic pathways. We also provide novel rationales for designing the next-generation cancer metabolism drugs.

## 1. Introduction

Uncontrolled, infinite proliferation is an essential characteristic of tumors. Therefore, recent studies highlight the differences in metabolic processes between cancer cells and their normal counterparts. In the 1920s, Otto Warburg found that unlike in normal cells, respiratory mechanisms are damaged in cancer cells, especially in the mitochondria. Cancer cells, therefore, cannot use oxidative phosphorylation (OXPHOS). Instead, they obtain ATP through glycolysis [1]. Even in oxygen-abundant environments, they are highly dependent on glycolysis (i.e., aerobic glycolysis). However, recent studies argue that the mitochondria of cancer cells remain intact and can produce energy using OXPHOS [2,3]. Despite this OXPHOS capability, many tumor types rely on aerobic glycolysis to supply enough building blocks for growth and adapt to hypoxic tumor microenvironments [4]. Tumors arise by mutations within oncogenes and tumor suppressor genes. These genetic mutations directly regulate the expression and activity of metabolic enzymes. For example, c-MYC activates glutamine uptake, and TP53 regulates lipid metabolism in cancer cells [5,6]. The abnormal metabolism of cancer cells is not merely a genetic mutation phenotype. It also directly affects tumor signal transduction pathways and cellular reactions. Based on this concept, the next-generation anticancer therapeutics examined in many studies and clinical trials target cancer-specific metabolic phenotypes. In this review, we discuss aberrant metabolic phenotypes of cancers and their roles in tumor progression. By analyzing interactions between metabolism and signaling pathways, we aim to establish potential therapeutic targets for new metabolism-based anticancer drugs.

## 2. Metabolic Characteristics of Cancers 

Genetic mutations confer the capability to bypass cell–cell contact inhibition and for the growth factor-orchestrated proliferation of cancer cells. However, poor vascularization in the tumor microenvironment induces chronic nutrient deprivation and reduced oxygen concentrations [7,8]. To survive and adapt to these harsh environmental stresses, cancer cells modify their metabolic pathways to capture external metabolites and maximize the efficiency of metabolic enzyme activities [9].

### 2.1. Glucose Metabolism

**Figure 1:**
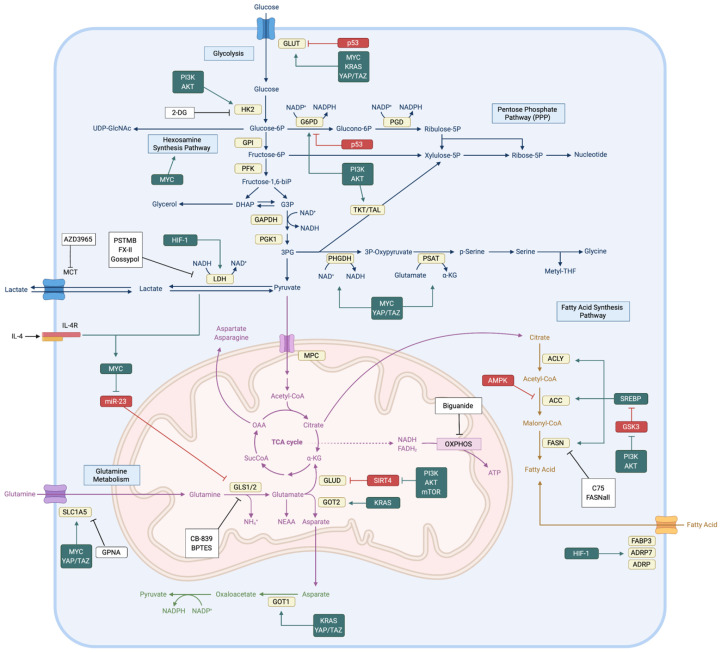
Interactions and inhibitors of cellular signaling and metabolism. Glucose, glutamine, and fatty acid metabolism are regulated by various types of oncogenic, tumor suppressive signaling. Oncogenic proteins (green), including PI3K/AKT, MYC, RAS, YAP/TAZ, and HIF-1, upregulate expression of nutrient transporters and metabolic enzymes (yellow). Tumor suppressive AMPK, miR-23, SIRT4, GSK3, and p53 inhibit metabolic processes (red). Some metabolism-targeting drugs (white) inhibit key metabolic steps, including glycolysis, NAD^+^ regeneration, fatty acid synthesis, and glutaminolysis. G6PD, glucose-6-phosphate dehydrogenase; PGD, phosphogluconate dehydrogenase; GPI, glucose-6-phosphate isomerase; PFK, phosphofructokinase; DHAP, dihydroxyacetone phosphate; G3P, glyceraldehyde 3-phosphate; GAPDH, glyceraldehyde 3-phosphate dehydrogenase; PGK1, phosphoglycerate kinase 1; 3PG, 3-phosphoglycerate; PHGDH, phosphoglycerate dehydrogenase; PSAT, phosphoserine transaminase; MCT, monocarboxylate transporter 1; MPC, mitochondrial pyruvate carrier; SucCoA, Succinyl-CoA; OAA, oxaloacetate; OXPHOS, oxidative phosphorylation; GSK3, glycogen synthase 3; HIF-1, hypoxia induced factor-1; FABP3, fatty acid binding protein 3; ADRP, adipose differentiation-related protein; SIRT4, sirtuin 4; GOT1/2, aspartate aminotransferase.

After the Warburg effect was revealed, studies have demonstrated that glucose metabolism is the key source to provide metabolic carbon in cancer cells [10]. When glucose enters the cytoplasm, it can be used as fuel by glycolysis, the hexosamine synthesis pathway (HSP), the pentose phosphate pathway (PPP), or the serine biosynthesis pathway. Each metabolic process provides precursors or intermediates (e.g., NADPH, nucleotides, pyruvate, amino acids, and methyl groups) for other metabolic pathways and cellular reactions. Therefore, the maintenance of stable glucose metabolism is an important requirement of cancer cell survival and cancer progression (Figure 1).

Glycolysis supplies various carbon intermediates and generates ATP and NADH. Oncogenic mutations have been shown to activate glycolytic enzymes. Glucose enters the cell via glucose transporter (GLUT) proteins. In the cytoplasm, glucose is phosphorylated by hexokinases (HKs) and remains trapped inside the cell. Through glycolysis, glucose is metabolized to the final product, pyruvate. During this process, the oncogenes c-MYC, KRAS, and YAP upregulate GLUT1 expression in cancer cells [11,12,13]. The overexpression of YAP and loss-of-function mutations in p53 increase GLUT3 expression, which causes its accumulation in the plasma membrane [14,15]. The phosphoinositide 3-kinase (PI3K)/AKT pathway is hyperactivated in cancer cells, and it upregulates HK2 activity by increasing mitochondrial HK association [16,17]. Cancer cells rely on aerobic glycolysis to fulfill metabolic requirements. As a result, lactate dehydrogenase (LDH) catalyzes pyruvate to lactate instead of acetyl-CoA, which can otherwise be used as TCA cycle intermediate. LDH has two isoforms, LDHA and LDHB. Both enzymes can catalyze both the pyruvate to lactate reaction and the reverse reaction. However, in various cancer cell lines, the LDHA isoform is highly expressed because LDHA prefers the pyruvate to lactate transition [18]. HIF-1a induces LDHA activation in hypoxic tumor microenvironments [19]. During this step, NADH, which is a byproduct of glycolysis, is oxidized to NAD^+^. NAD^+^ regeneration maintains glycolysis.

Glucose-6-phosphate (G6P) is produced by HK. G6P can initiate the PPP, which has two distinct phases, oxidative and non-oxidative. The oxidative phase produces NADPH and ribulose-5-phosphate (R5P); the non-oxidative phase produces only R5P, the precursor for nucleotides [20]. NADPH is necessary for fatty acid synthesis. NADPH also has reducing power, which is required to regenerate glutathione (GSH) from glutathione disulfide (GSSG). Given that cancer cells are exposed to chronic metabolic stress, control of cellular reactive oxygen species (ROS) levels is important to avoid negative outcomes including apoptotic cell death [21]. Hence, the PPP is critical for maintaining redox homeostasis and protecting cancer cells from oxidative stress. Several oncogenic proteins upregulate PPP influx. In cancer cells, hyperactive PI3K/AKT and mTORC1 signaling increase the expression of rate-limiting enzymes in the PPP (e.g., G6PD and RPIA) via sterol regulatory element-binding protein 1 (SREBP1) activation [22]. Aberrant AKT activation in cancer cells activates the transketolase enzyme in the PPP via direct phosphorylation [23]. Oncogenic c-Myc upregulates PPP influx [24]. Finally, PPP activity increases when the loss-of-function mutation of p53 cannot bind and inhibit G6PD [25].

The HSP provides UDP-GlcNAc, which has an important role in protein post-translational modifications, such as glycosylation. Because GlcNAcylation regulates protein stability, GlcNAcylation of p53 protects the protein from phosphorylation-mediated protein degradation [26]. mTORC1/MYC hyperactivation increases HSP activation in breast cancer cells by upregulating O-GlcNAc transferase (OGT) [27].

The serine biosynthesis pathway diverges from the glycolysis pathway at the level of 3-phosphoglycerate (3-PG). Phosphoglycerate dehydrogenase (PHGDH) is the first enzyme of the serine biosynthesis pathway and is the rate-limiting enzyme that converts 3-PG to 3-phosphohydroxypyruvate. During this metabolic process, phosphoserine aminotransferase (PSAT1) generates α-ketoglutarate (α-KG) as a reaction byproduct. Thus, the serine biosynthesis pathway can provide a TCA cycle intermediate for further ATP production and anabolic metabolism [28]. The final products of the serine synthesis pathway (i.e., serine and glycine) are precursors for other metabolic pathways, including folate metabolism and methionine metabolism. NADPH and methyl groups are generated via folate and methionine metabolism. Consequently, in addition to amino acids, the serine biosynthesis process produces reducing potential and methyl groups that are involved in post-translational modification and epigenetic regulation [29]. PHGDH is genetically and transcriptionally upregulated in diverse cancer cell lines. The PHGDH gene is amplified and enzyme expression is elevated, especially in breast cancer cells [30,31]. c-Myc transcriptionally upregulates PHGDH and other serine biosynthesis enzymes, including PSAT1, phosphoserine phosphatase (PSPH), and serine hydroxymethyltransferase 1 (SHMT1) [32]. Therefore, glycolytic intermediates can branch off into key biosynthetic pathways to generate nucleotides, amino acids, and fatty acids that are essential to meet the increased biosynthetic needs of cancer cells.

### 2.2. Glutamine Metabolism

Glutamine is the most abundant amino acid in plasma. It supplies carbon and nitrogen by participating in various cellular reactions. Glutamine is critical for cancer cell proliferation because nitrogen is an essential metabolite for nucleotide biosynthesis and glutamine is a precursor for synthesis of other non-essential amino acids (NEAAs) and fatty acids [33] (Figure 1).

Glutamine is imported into the cytoplasm via the glutamine transporter, ASCT2 (i.e., SLC1A5). Because glutamine is the major nitrogen donor, various oncogenic signals promote ASCT2 activity. For example, oncogenic c-Myc and n-Myc upregulate ASCT2 expression via ATF4 in neuroblastoma cells and promote glutamine uptake [5,34,35]. mTORC1 induces ASCT2 expression [36]. Tumor microenvironment factors promote glutamine transporter expression. The inflammatory cytokine, IL-4, binds its receptor and induces c-Myc transcription in breast cancer cells [37,38]. Lactate is the final product of aerobic glycolysis. It is imported by MCT-1 transporter and stabilizes c-Myc to increase ASCT2 expression [39]. Ras-transformed cancer cells use extracellular vesicles to capture external plasma proteins via micropinocytosis [40,41].

Imported glutamine has two possible fates. First, glutamine can serve as the nitrogen donor for amino acid and nucleotide biosynthesis. Glutaminase (GLS1/2) enzymes catalyze glutamine to glutamate and produce ammonia (NH_3_). Glutamate can also be converted to the TCA cycle intermediate, α-KG, and produce NH_4_^+^. This ammonia group can be transferred via carbamoyl phosphate synthetase and phosphoribosyl pyrophosphate synthase, which are rate-limiting enzymes of the pyrimidine and purine synthesis pathways, respectively [42]. Nitrogen from glutamine is a substrate for transaminases to synthesize NEAAs (e.g., alanine, asparagine, and serine). The microRNAs miR-23a and miR-23b inhibit GLS activity by targeting the 3′-UTR region of GLS mRNA. However, in cancer cells, c-Myc activation inhibits miR-23a/b and promotes GLS translation [43]. Moreover, PI3K/AKT axis and downstream mTOR pathway hyperactivation induce glutamate dehydrogenase (GLUD) expression by inhibiting SIRT4. This inhibition PARylates (ADP-ribosylation) and inhibits GLUD activity [44,45]. Conversely, in pancreatic cancer cells, KRAS mutation downregulates GLUD activity and upregulates the aspartate synthase (GOT1) enzyme to provide NADPH for the proper maintenance of redox homeostasis [46].

The second fate of imported glutamine occurs when it serves as a carbon donor for fatty acid synthesis. Because the plasma membrane and subcellular organelles consist of lipid bilayers, de novo lipid biosynthesis is required for cell proliferation. Hence, lipid biosynthesis is upregulated in various types of cancers (e.g., prostate, lung, and stomach) [47,48]. Under normoxic conditions in normal cells, most acetyl-CoA, which is a precursor for fatty acid synthesis, comes from glycolysis. However, because of aerobic glycolysis, cancer cells usually transform pyruvate to lactate rather than acetyl-CoA. Under these circumstances, most of the acetyl-CoA in cancer cells is acquired from the glutamine-TCA cycle axis [49,50]. Glutamine is catalyzed to α-KG, which then enters the anabolic phase of the TCA cycle to produce citrate. Citrate translocates from the mitochondria to the cytoplasm and is catalyzed to acetyl-CoA by the ATP citrate lyase (ACLY) enzyme. Thereafter, fatty acid synthase (FASN) mediates long-chain fatty acid synthesis.

Glutamine fuels TCA cycle intermediates, which are emerging as mediators of malignant transformation in cancer. As a result of aerobic glycolysis and OXPHOS, glutamine becomes the principal source of NADH and FADH_2_ in cancer cells [51]. However, OXPHOS inevitably produces ROS that might induce DNA damage and oxidative stress [52]. Hence, oncogenic mutation induces diverse NADPH-providing mechanisms that confer sufficient reducing power to manage ROS levels [53].

### 2.3. Fatty Acid Metabolism

Recent studies underscore the importance of fatty acid metabolism in cancer progression. Fatty acids not only have roles as structural components but are also secondary messengers (DAG and IP_3_). Thus, fatty acid synthesis is vital for cellular response and proliferation. Fatty acid synthesis requires significant amounts of NADPH, which is crucial for redox homeostasis. Therefore, it is regulated by various signal pathways to maintain the balance between redox homeostasis and cell growth (Figure 1).

Fatty acid synthesis is coordinated by SREBPs, which are transcription factors for lipid biosynthesis enzymes. SREBPs are synthesized and sequestered in the endoplasmic reticulum as inactive precursors [54]. When cellular lipid levels are low, Golgi-associated MBTPS1/2 protease cleaves SREBPs at the N-terminus. The cleaved product is translocated into the nucleus, where it binds to the SRE protein and induces target gene expression [55]. In the nucleus, GSK3β inhibits SREBP stability via FBXW7-mediated ubiquitination [56]. However, aberrantly activated PI3K/AKT and mTORC2 signaling inhibit GSK3β to allow for the higher expression of SREBP in various cancer cell types [57]. As a direct target gene of SREBP, ACLY enzyme converts citrate to acetyl-CoA at the very first step of fatty acid synthesis. E3 ligase UBR4 and Cullin3-KLHL25 ubiquitinate and destabilize ACLY [58]. ACLY acetylation via p300 inhibits ubiquitination and increases its stability [59]; SIRT2 deacetylates and destabilizes ACLY [59]. After ACLY produces acetyl-CoA, acetyl-CoA carboxylase (ACC) converts acetyl-CoA to malonyl-CoA. ACC is a well-known target for AMP-activated protein kinase (AMPK), a master regulator of energy homeostasis. However, some lung adenocarcinoma cell lines have mutant liver kinase B1 (LKB1), which is an upstream regulator of AMPK. These cell lines have a constitutively active state of ACC and increased fatty acid synthesis [60].

Fatty acid synthesis is an oxygen-consuming process. Therefore, cancer cells try to compensate for fatty acid synthesis by upregulating external lipid uptake instead of using de novo fatty acid synthesis. This upregulation especially occurs during metabolically challenging situations (e.g., hypoxia or nutrient deprivation). In breast cancer cells, HIF-1α promotes fatty acid uptake by increasing expression of the fatty acid-binding receptor proteins FABP3, FABP7, and ADRP [61]. KRAS activation facilitates macropinocytosis, which promotes extracellular lipid uptake and lysosomal degradation [62]. Cancer cells use these mechanisms to overcome metabolic hurdles that restrict metabolite synthesis.

## 3. Oncogenic Signal Pathways Regulate Cancer Metabolism

Studies have found significant interactions between oncogenic signaling and aberrant metabolic phenotypes. Genetic mutations in oncogenes and tumor suppressors directly regulate transcription of metabolic enzymes or indirectly regulate enzyme activity via management of regulatory factors such as post translational modifications (PTM) and feedback loops. These complex interactions between signaling and metabolism confer metabolic plasticity and enable cancer cells to adapt to severe metabolic stress environments (Table 1).

### 3.1. Hippo Pathway

The Hippo pathway controls cell proliferation, organ size, and tissue homeostasis. It consists of MAPK family, the Ste20-like kinase (MST) 1/2, large tumor suppressor kinase (LATS) 1/2 core kinases, and the transcriptional co-activators YAP and TAZ [97,98,99,100,101,102]. When the pathway is activated, upstream signals phosphorylate and activate MST1/2 and LATS1/2, thereby phosphorylating YAP/TAZ. Consequently, YAP/TAZ are sequestered in the cytoplasm via 14-3-3 proteins and are degraded by E3 ligase β-TRCP [103,104,105]. However, when Hippo signaling is turned off, YAP/TAZ translocate into the nucleus and bind with transcriptional enhanced associate domain (TEAD) transcription factors to induce oncogenic target gene expression. The Hippo pathway is regulated by a wide range of upstream regulators, including cell–cell contact, mechanical cues from the surrounding environment, Wnt signaling, G protein-coupled receptor (GPCR)–ligand interactions, and various cellular stress conditions [106,107,108,109]. YAP/TAZ activity is hyperactive in many types of cancers [110,111].

As a master regulator of cell growth, the Hippo pathway is involved in multiple metabolic processes. YAP/TAZ activity promotes glycolysis by directly and indirectly enhancing glycolytic enzyme activity. In particular, YAP/TAZ activity increases GLUT3 expression by TEAD and induces HK2 expression by FOXC2 [63,64]. YAP/TAZ also promote LncRNA BCAR4 expression and upregulate HK2 and PFKFB3 via Hedgehog signaling [65]. YAP/TAZ also enhances glutamine metabolism, increasing glutamine transporter SLC1A5 and SLC7A5 expression in breast cancer cells [66,67]. YAP/TAZ and TEAD upregulate glutamine and amino acid metabolism by expressing amino acid transporters [112]. YAP/TAZ induce glutaminase and transaminase expression, including GOT1 and PSAT1, which produces NEAAs and TCA cycle intermediates [68,69]. YAP/TAZ accumulate lipids and directly modulate bile acid components, which enhances the metastatic potential of cancer cells [70].

Cell cycle progression and cell proliferation are determined by nutrient concentrations. Metabolic conditions highly affect the Hippo pathway. Glucose metabolism has significant effects on YAP/TAZ activity. High glucose levels increase glucose flux to the HSP, which produces UDP-GlcNAc used for glycosylation. Thus, abundant glucose induces YAP O-GlcNAcylation and interferes with the LATS and βTrCP interaction, which in turn enhances YAP/TAZ activity. YAP is hyperactivated by O-GlcNAcylation in pancreatic and liver cancers [113,114]. Glycosylation of LATS2 inhibits its activity by interfering with the LATS2 and MOB1 interaction in breast cancer [115]. In contrast, energy stress induced by glucose deprivation inhibits YAP/TAZ activity via Hippo-dependent and -independent mechanisms. The energy sensor AMPK is activated as ATP decreases. AMPK directly phosphorylates YAP at serine 61 and serine 94, which blocks the YAP-TEAD interaction [15,116]. AMPK indirectly inhibits YAP via AMOTL1 phosphorylation and activation [117].

External hormone levels can regulate the Hippo pathway. Lipid hormones, such as lysophosphatidic acid and sphingosine-1-phosphate, inhibit the pathway through GPCRs [109,118]. The peptide hormone glucagon, which increases blood glucose levels, activates the Hippo pathway through cAMP and PKA activation [119]. Subcellular lipid components can also regulate Hippo pathway kinases. SREBP is an upstream regulator of the Hippo pathway. When SREBP is activated, the mevalonate pathway enhances geranylgeranylation of RhoA GTPase. RhoA is an F-actin cytoskeleton regulator, and F-actin is a well-established upstream factor of LATS kinase. Therefore, increased fatty acid metabolism in cancer cells aberrantly activates RhoA and enhances YAP/TAZ activity by inhibiting LATS [120,121]. Interestingly, YAP/TAZ are hardly expressed in hematologic malignancies, and the forced expression of YAP has been shown to mediate tumor suppressive functions [122,123]. It will be important to elucidate Hippo-YAP pathway-induced aberrant metabolism in blood cancers, including leukemia and lymphoma.

### 3.2. PI3K-AKT/mTOR Pathway

In normal cells, cell proliferation and anabolic metabolism are delicately regulated by external growth factors (e.g., insulin and hormones). Mitogen signals activate signal transduction pathways (e.g., the PI3K-AKT and mTOR pathways) to induce cell cycle progression. The PI3K-AKT pathway is activated by receptor tyrosine kinases (RTKs) and GPCR signaling. When RTKs or GPCRs are activated, PI3K phosphorylates the phospholipid PIP_2_ to PIP_3_. However, the tumor suppressor protein, PTEN, dephosphorylates PIP_3_ to PIP_2_ and thus inhibits PI3K/AKT signaling. Gain-of-function mutation of PI3K and loss-of-function mutation of PTEN are found in various cancer types [124]. After PIP_3_ accumulates, PIP_3_ binds to and activates AKT via PDK1- and mTORC2-mediated phosphorylation [125,126]. Homeostasis between active and inactive AKT governs cell proliferation, metabolic adaptation, and tumorigenesis.

Notably, AKT promotes mTORC1 activity through inhibitory phosphorylation of TSC2; this change inhibits RHEB GTPase [71]. mTORC1 phosphorylates ribosomal S6K and 4E-BP1 and turns on the cellular protein translation machinery [127]. After phosphorylation, S6K unwinds the secondary structures of mRNAs and starts translation through elF4B-elF4A heterodimerization [78,128,129]. 4E-BP phosphorylation dissociates 4E-BP from elF4E and enhances accessibility of the translation initiation complex to the 5′ region of mRNAs [130]. Therefore, mitogen-activated AKT promotes protein production through mTORC1.

PI3K/AKT signaling regulates metabolic enzymes. Upon activation by insulin, AKT inhibits the glycogen synthesis pathway by phosphorylating GSK3 [72. Thus, PI3K/AKT is an important signal transduction pathway in glucose metabolism. PI3K/AKT upregulates glycolysis directly and indirectly through post-translational modifications (e.g., phosphorylation, glycosylation) of metabolic enzymes. For example, AKT phosphorylates and activates AS160 protein, which enhances membrane trafficking of GLUT [73,74,75]. AKT directly inhibits TXNIP via inhibitory phosphorylation and increases membrane expression of GLUT1/4 by inhibiting endocytosis [76]. AKT also increases the efficiency of glycolytic enzymes, including HK2 and PFK1. AKT phosphorylates and activates HK2 by increasing mitochondrial integration of HK2 [16,17]. The activity of PFK1, which is regulated by fructose-2,6-bisphosphate (F-2,6-BP), is increased by AKT. AKT phosphorylates PFKFB; this change catalyzes fructose-6-phosphate to F-2,6-BP and thereby increases the productivity of glycolysis [131]. AKT signaling also promotes glycolysis via HIF-1α transcription factor. HIF-1 activates some glycolytic components, including GLUT. LDH induction by HIF-1 promotes lactate secretion and aerobic glycolysis [77].

PI3K/AKT signaling upregulates glutamine and fatty acid metabolism. AKT increases Myc translation via mTORC1 and inhibits Myc degradation through GSK3 and FOXO3A inhibition [78,79,80]. Because MYC regulates glutamine metabolism, the activation of MYC increases the cellular nitrogen supply. AKT thus promotes nucleotide synthesis [132]. Cancer cells require de novo lipid synthesis to maintain and supply membrane components necessary to continue infinite cell division. AKT affects the de novo lipid biosynthesis process via direct phosphorylation of ACLY, the very first enzyme of lipid synthesis, and thus enhances enzyme efficiency [81]. AKT increases histone acetylation in various cancer types through ACLY regulation [133]. AKT-induced mTORC1 activation enhances the translation and cleavage processing of SREBP family transcription factors [82,83]. AKT-mediated GSK3 inhibition further prevents SREBP1 degradation [134]. Therefore, understanding the metabolic reprogramming via PI3K/AKT signaling will elucidate effective therapeutic alternatives to PI3K inhibitors for cancer treatment.

### 3.3. Myc Pathway

Transcription factor c-Myc regulates gene expression with MAX and is one of the most hyperactivated genes in cancer cells [135,136]. Because Myc is at the cross-points of growth-related signaling processes (e.g., EGFR, AKT, and GPCRs), delicate regulatory mechanisms modulate Myc expression and transcriptional activity. Growth factors upregulate Myc expression via the Wnt/β-catenin and RAS/ERK pathways [137,138]. mTOR, which is activated by AKT, also increases translation of Myc [79,128]. Cell cycle checkpoint proteins regulate Myc activation. For example, transcription factor FOXO3A activates p19^ARF^, which then directly binds and inhibits the transcriptional activity of Myc [139,140]. AKT directly phosphorylates and downregulates FOXO3A activity [141]. Thus, AKT can indirectly activate Myc. However, cancer cells can bypass these regulatory mechanisms via genetic alterations and aberrant signal pathways. Myc family transcription factor genes, including c-Myc, n-Myc, and l-Myc, are amplified in leukemia, colon cancer, neuroblastoma, and non-small cell lung cancer cells [142,143,144,145,146]. In Burkitt’s lymphoma, Myc genes are translocated to the highly active promoter and enhancer-proximal regions [147,148]. Aberrant activation of Wnt/β-catenin and ERK hyperactivate Myc expression in colorectal cancer and melanoma [149,150]. These bypass mechanisms increase cellular Myc protein levels and promote chromatin accessibility and transcriptional activity of Myc.

Myc regulates expression of genes associated with glucose, glutamine, and fatty acid metabolism. Myc enhances metabolic rewiring of cancer cells through upregulation of glycolysis and glutaminolysis. Myc induces many glycolytic enzymes (e.g., GLUT, HK2, and PFK) that catalyze the commitment steps during glycolysis [11,84]. In cancers, regeneration of NAD^+^ is required to maintain glycolytic capacity. Myc upregulates transcription of LDH and MCT1 enzymes and promotes regeneration of NAD^+^ [85,86]. Because Myc increases cellular glucose uptake, it is not surprising that Myc also elevates PPP and HSP influx. Myc upregulates the rate-limiting enzymes of the PPP, including G6PD and TKT [87]. In breast cancer cells, Myc promotes O-GlcNAc transferase activity through HSP90-mediated protein stabilization [24,27]. Glucose metabolism also modulates post-translational modification of Myc as a feedback regulatory mechanism. During glucose deprivation, decreased O-GlcNAcylation destabilizes Myc protein levels [151].

Glutamine is extensively studied as a critical carbon and nitrogen source. As a master regulator of glutamine metabolism, Myc upregulates aspects of glutamine metabolism from uptake to anaplerotic reactions. Myc transcriptionally induces glutamine transporters SLC1A5 and SLC38A5 [5]. Myc also activates GLS expression, which mediates glutamine to glutamate conversion via inhibition of miR-23 [43]. Myc increases TCA cycle intermediates (e.g., α-KG and ammonia groups) for NEAA synthesis, through activation of GLUD and other transaminases [34,88]. Using ^13^C isotope labeling, studies found that glutamine serves as the major carbon source during the TCA cycle and fatty acid synthesis when aerobic glycolysis blocks glucose entrance to the TCA cycle influx [152]. Acetyl-CoA is generated using citrate and is the initial metabolite of fatty acid synthesis. Thus, Myc indirectly upregulates fatty acid synthesis by increasing glutamine-mediated TCA cycle influx [153]. Myc is also regulated by glutamine metabolism. When glutamine is restricted, subsequent nitrogen depletion represses the transcription machinery. Unstable transcription induces DNA damage and genomic instability. Therefore, glutamine deprivation induces p53 phosphorylation and transcribes several micro RNAs targeting the 3′-UTR region of Myc, including miR-34b/c and miR-145 [154,155,156]. Therefore, deregulated MYC elicits dependence on MYC-driven metabolic pathways, such that a reliance on specific metabolic enzymes provides novel anticancer drug targets.

### 3.4. p53 Pathway

p53 is one of the most well-known tumor suppressor genes that affect cellular processes including apoptosis, cell cycle progression, and metabolism. p53 is activated during cellular stress conditions such as DNA damage and nutrient deprivation. It determines whether adaptation or cell death occurs, depending on the types and intensities of the stressors [157]. p53 is modulated at transcriptional, translational, and post-translational levels. The stability of p53 is regulated by E3 ligase MDM2 [158,159]. AMPK phosphorylates, acetylates, and consequently stabilizes p53 under energy stress conditions [160,161]. By upregulating MDM2 to promote p53 degradation, p53 induces a negative feedback loop to maintain homeostasis [157]. Studies found that p53 halts cell cycle progression to repair DNA damage before restarting cell proliferation [162]. Moreover, p53 inhibits cancer cell migration, angiogenesis, tumorigenesis, and the metabolic reprogramming of various cancers [157].

p53 reduces aerobic glycolysis and upregulates mitochondrial catabolic processes, including fatty acid oxidation (FAO) and OXPHOS. p53 transcriptionally represses GLUT1 and GLUT4 expression [89]. It also induces the TP53-inducible glycolysis and apoptosis regulator (TIGAR) and inhibits PFK1, the rate-limiting enzyme of glycolysis, by lowering its allosteric activator fructose-2,6-bisphosphate (F-2,6-BP) [90]. p53 increases TCA cycle influx via LDH inhibition and activation of pyruvate dehydrogenase (PDH) [91]. It also indirectly inhibits several glycolytic enzymes, including HK and glucose-6-phosphate isomerase, via miR-34a [163]. To reprogram the metabolic process from the biomass-producing anabolic phase to the energy-generating catabolic phase, p53 increases glutaminolysis via transcriptional induction of GLS2 and FAO [164,165,166]. p53 induces carnitine palmitoyltransferase 1 (CPT1) transcription and phosphatidate phosphatase LPIN1 activation to increase mitochondrial fatty acid uptake and FAO related gene expression, respectively [6,92]. p53 directly or indirectly accumulates NADH and FADH2 by enhancing catabolic metabolism, and thus activates the electron transport chain and OXPHOS. To support this metabolic transition, SCO2 expression by p53 facilitates electron transport chain capacity [93]. In almost one-half of cancers, the mutation of p53 contributes to aerobic glycolysis and anabolic metabolism [167]. However, findings also suggest that in some subtypes of hepatocellular carcinoma, wild type p53 induces p53 upregulated modulator of apoptosis (PUMA) expression and inhibits mitochondrial pyruvate uptake by inhibiting MPC transporter. Consequently, p53 inhibits OXPHOS and increases aerobic glycolysis dependency [168]. Although p53 has long been shown to play key roles in DNA damage, cell cycle, and oncogenic activation, the metabolic pathways regulated by p53 and their cooperation in controlling cancer metabolism will provide critical aspects to treat cancer.

### 3.5. LKB1/AMPK Pathway

AMPK is a well-conserved energy sensing kinase. AMPK inhibits anabolic pathways and promotes catabolic cellular processes. Hence, AMPK activation allows cells to withstand cellular energy stress conditions. Unlike other metabolic proteins, the regulation of AMPK depends on cellular concentrations of AMP and ADP but is independent of other metabolic intermediates. Increases in AMP/ATP ratios elicit AMP interaction, followed by conformational changes in AMPK. Consequently, cellular energy stress-mediated AMPK conformational change allows upstream kinases to phosphorylate the AMPK alpha subunit [169,170]. AMPK is also modulated by growth factor-mediated GPCR activation and cellular calcium levels. As an upstream kinase, LKB1 phosphorylates the AMPK alpha subunit during energy stress and CAMKK phosphorylates AMPK alpha by sensing calcium concentrations [171,172]. In the context of cancer, AMPK activation by stress environments, including hypoxia and energy or nutrient deprivation, confers stress resistance characteristics in various cancers [173].

Whether AMPK is an oncogenic protein or a tumor suppressive protein remains to be determined. In clinical settings, AMPK activators including metformin, a type 2 diabetes drug, and AICAR, are used to inhibit cancer cell proliferation [174,175]. Crosstalk between the Hippo and mTOR pathways is the mechanism underlying its anti-proliferative function. In a nutrient-deprived environment, AMPK inhibits YAP-TEAD interactions via LATS kinase or via direct phosphorylation of YAP [15,116,117]. AMPK inhibits mTORC1, which is a key regulator of protein translation machinery, via direct phosphorylation of TSC2 and RAPTOR [176,177]. However, AMPK also acts as an oncogenic protein by promoting anoikis resistance, migration, and metastasis. For example, AMPK promotes cell survival in prostate cancer and facilitates cell migration and metastasis via the androgen receptor-CAMKK axis [178,179]. AMPK also confers anoikis resistance via mTORC1 inhibition [180].

ACC is a well-established target of AMPK. ACC mediates conversion of acetyl-CoA to malonyl-CoA during de novo fatty acid synthesis. AMPK reduces ATP consumption through inhibitory phosphorylation of ACC [94]. Decreased fatty acid synthesis flux by AMPK reduces NADPH consumption [181]. To meet the cellular requirements of lipid metabolites, AMPK maintains lipid homeostasis by upregulating fatty acid uptake via CD36 and other lipid transporters [95,96]. Protein translation is highly energy-consuming and uses diverse anabolic pathways, including the PPP and glutaminolysis. AMPK allows cancer cells to adapt to harsh nutrient conditions, such as glucose deprivation, by inhibiting mTORC1 and the translation machinery [173]. Therefore, in addition to AMPK being a well-accepted target for the treatment of metabolic syndrome and type 2 diabetes, recent evidence suggests AMPK as a key metabolic target in cell growth and tumorigenesis.

## 4. Role of Aberrant Metabolic Phenotypes in Cancer Biology

Previously described metabolic phenotypes of cancer, such as aerobic glycolysis, increased glutamine, and fatty acid anabolic metabolism, are results of deregulated oncogenic and tumor suppressive signal transduction pathways. However, studies indicate that metabolic rewiring of cancer is not merely an outcome of aberrant signal pathways, but it also offers potential benefits for cancer cell proliferation and metastasis. Therefore, understanding metabolism-induced tumor phenotypes is important for establishing strategies to treat cancer.

### 4.1. Redox Homeostasis

Altered metabolic pathways lead to the increased building block synthesis and bioenergetic pathways that are critical for cancer cell proliferation. However, these metabolic pathways contain numerous oxidative and reductive processes that inevitably generate ROS [182]. To some extent, ROS promote tumor progression by inducing genomic instability. However, uncontrolled ROS accumulation activates cell death signals [51,183,184,185]. After being triggered, superoxide dismutase proteins transform ROS to H_2_O_2_, which is detoxified by peroxiredoxins (PRXs) or glutathione. Oxidized PRXs and glutathione disulfide are recycled by thioredoxin reductase and glutathione reductase, respectively [186]. Both recycling metabolisms require NADPH as the reducing agent. Therefore, cancer cells upregulate NADPH-producing metabolic processes and antioxidant enzymes to maintain redox homeostasis.

**Figure 2:**
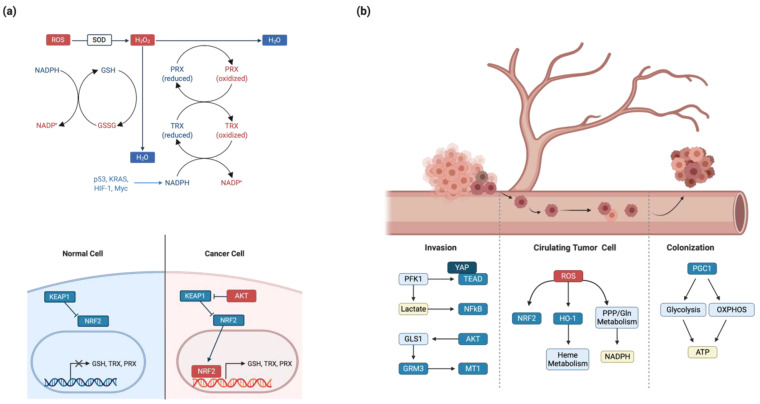
Cancer metabolism promotes redox homeostasis during metastasis. (**a**) Oncogenic signaling, such as KRAS, HIF-1, and MYC, activates NADPH-producing metabolic processes. NADPH provides reducing power for recycling antioxidant metabolites. (top) Aberrant NRF2 activation in cancer cells upregulates expression of redox metabolites and enzymes. (bottom) (**b**) Cancer activates various metabolic enzymes and oncogenic signaling during each step of the metastatic cascade. YAP/TEAD, NF-κB, and AKT signaling induces invasion by enhancing metalloprotease expression. Redox homeostasis-related metabolic processes are increased during circulating tumor cell (CTC) formation. NRF2, HO-1 enhances antioxidant enzymes and heme metabolism. The PPP and glutamine metabolism facilitate NADPH accumulation. During the colonization step, PGC1 increases metabolic plasticity and activates ATP-generating pathways. SOD, superoxide dismutase; NADP, nicotinamide adenine dinucleotide phosphate; GSH, glutathione; GSSG, glutathione disulfide; PRX, peroxiredoxin; TRX, thioredoxin reductase; GRM3, metabotropic glutamate receptor 3; MT1, metallothionein 1; HO-1, heme oxygenase.

Cancer cells upregulate Nuclear factor erythroid 2-related factor 2 (NRF2) activity to maintain redox homeostasis, which is a master regulator of the cellular oxidative stress response. In the normal state, Kelch-like ECH-associated protein 1(KEAP1) ubiquitinates NRF2 via Culin 3-dependent E3 ligase complex and promotes NRF2 degradation. However, a hypoxic environment or oxidative stress dissociates the KEAP1-NRF2 interaction and thereby promotes NRF2 activity [187,188,189]. Oncogenic signaling (e.g., K-Ras, B-Raf, and Myc) increases Nrf2 activity via KEAP1 inhibition [190]. In non-small cell lung cancer, mutant KEAP1 fails to inhibit NRF2 function [191]. KEAP1 is transcriptionally inhibited by hypermethylation in 71% of invasive breast cancers [192]. NRF2 activation induces expression of antioxidative enzymes that synthesize glutathione, TRX, PRXs, and the NADPH synthesis machinery (Figure 2a).

Oncogenic mutation upregulates NADPH production. For example, p53 mutation promotes glucose uptake but inhibits glycolysis by upregulation of GLUT1 and GLUT4 and inhibition of TIGAR, respectively. Therefore, p53 drives glucose flux toward the PPP. Given that the oxidative phase of the PPP is the major NADPH-generating metabolic pathway, p53 indirectly promotes cellular redox homeostasis [89,90]. In pancreatic cancer, KRAS G12D driver mutation elevates glutamine-based NADPH synthesis. After glutamine is catalyzed to glutamate by GLS, KRAS activates the transaminase GOT1/2 instead of glutamine synthetase GLUL. Transaminases convert glutamate to aspartate. In the cytosol, aspartate is transformed to oxaloacetate and malate by GOT1 and MDH1, respectively. Thereafter, malic enzyme generates NADPH by converting malate to pyruvate [45]. Hypoxic microenvironment-induced HIF-1α activation also increases NADPH synthesis. HIF-1α upregulates glycolysis and promotes NADPH production via the PPP [77]. Myc also enhances NADPH synthesis via serine synthesis and one carbon metabolic pathways. SHMT1/2 and MTHFD1/2 enzymes mediate the rate-limiting step of one carbon metabolism and are direct targets of Myc. In cancer, dysregulated Myc expression increases the activities of these enzymes and thereby contributes to the NADPH production that is a byproduct of methylene-THP synthesis [193,194]. NADPH is used to recycle oxidized antioxidant metabolites, including Glutathione and PRXs [195]. Synthesis of these metabolites is also hyperactivated by oncogenic signaling. Glutathione consists of glutamate, cysteine, and glycine. In many cancer types, deregulated Myc increases GLS enzyme activity and thus enhances glutamate synthesis [43]. The cysteine transporter, xCT antiporter, is hyperactivated in various cancers [196,197]. AKT also accumulates glutathione via NRF2 activation [198]. However, the contribution of ROS to cancer development remains controversial and is clearly highly complex [199]. Hence, understanding the cellular metabolism that governs ROS-related signaling will provide valuable insights to target cancer cells.

### 4.2. Invasion and Metastasis

Metabolic alteration that occurs via deregulated oncogenic signaling pathways affects cancerous phenotypes such as invasion and metastasis. Metastasis is the main cause of death in patients with cancer [200]. Secondary tumors are more aggressive than their primary counterparts because they experience colonization and proliferation under harsh environmental cues. Cancer metastasis consists of the five steps of invasion, dissemination, circulating tumor cells, colonization, and secondary tumor formation. Each step requires unique metabolic transitions to adapt to the surrounding environment [201,202] (Figure 2b).

The first step of metastasis, invasion is the infiltration of cancer cells into neighboring tissues. Invasion requires extracellular matrix (ECM) degradation by metalloproteases, inhibition of cell-cell contact, and activation of epithelial-mesenchymal transition (EMT) genes [203,204]. Although most cancers arise from epithelial cells, cancer cells change their cell phenotypes from epithelial to mesenchymal during the invasion stage to avoid cell-cell contact [205]. Glycolysis has an important role during invasion. The rate-limiting enzyme PFK1, which regulates the first committed step of glycolysis, directly binds to YAP and activates the YAP–TEAD interaction [206]. Hence, PFK1 promotes the YAP-TEAD target gene transcription involved in cell migration and EMT induction [207]. The final product of aerobic glycolysis, lactate, is secreted via MCT transporter that consequently acidifies the tumor microenvironment. Acidic conditions activate NF-κB signaling, which leads to metalloprotease gene transcription and tumor invasion [208]. Metabolic activity-driven ROS production triggers PPAR- γ coactivator 1 (PGC-1), which functions as a master regulator of mitochondrial biogenesis. PGC-1 promotes or inhibits invasion in a context-dependent manner. In prostate cancer, PGC-1 inhibits metastasis via estrogen-related receptor alpha (ERRα)-mediated transcriptional regulation. In breast cancer, PGC-1 enhances invasion and confers drug resistance [209,210,211]. Similarly, glutamine metabolism promotes invasion. The PI3K-AKT pathway upregulates GLS enzyme expression. In the tumor microenvironment, secreted glutamate acts as a paracrine ligand for GRM3 GPCR, which induces MT1 metalloprotease translocation to the plasma membrane via Rab27 GTPase [212]. Inhibition of ACC by AMPK-mediated phosphorylation accumulates acetyl-CoA in the cytoplasm. Because acetyl-CoA confers acetylation potential, it thereby induces the SMAD2 acetylation that eventually upregulates EMT genes [213]. However, recent studies highlight EMT-independent cancer progression [214]. It will be important to elucidate the metabolic rewiring, which are responsible in the EMT transcription factor-independent tumor development.

After invasion and dissemination, metastatic cancer cells enter the blood vessels. As a result of blood pressure-induced shear stress and loss of cell-to-ECM adhesion, survival in the blood stream is one of the biggest obstacles encountered during metastasis [201]. In normal cells, loss of anchorage between the cell and the ECM increases ROS and induces anoikis. To avoid anoikis, cancer cells upregulate antioxidant mechanisms to eliminate ROS [215]. NRF2 transcription factor promotes transcription of antioxidant genes in various cancer cells. For example, NRF2 upregulates HO-1 via Bach1 and enhances metastasis by increasing antioxidant enzymes and metabolites [216,217,218]. NADPH-generating pathways, including the PPP and glutamine metabolism, are also highly expressed by ERBB2 signaling to confer ROS resistance [219,220]. After moving to the metastatic organ, cancer cells penetrate and colonize the secondary site (i.e., micrometastasis). Cancer cells maximize ATP production during colonization and secondary proliferation. PGC-1α activation confers metabolic plasticity. Consequently, these metabolic plasticities enable cancer cells to adapt to the external nutrient conditions and produce ATP via both glycolysis and OXPHOS [221]. In addition to glucose and glutamine, fatty acids can be the fuel for ATP synthesis. Hyperactivated CD36 fatty acid transporter increases cellular lipid catabolism, including β-oxidation in HER2-positive breast cancers [222].

### 4.3. Cancer Stemness

Cancers are highly heterogenous, such that each cancer cell has different metabolic or genetic phenotypes even within the same tumor mass [223]. Among diverse subtypes of cancer cells, a small subset possesses self-renewal and tumor-initiating capacities. These stem cell-like cancer cells are called cancer stem cells (CSCs) [224]. Unlike in proliferating cancer cells, in a heterogenous population, CSCs have slower cell cycles and are highly dedifferentiated. Although <1% of cancer cells are known to be CSCs, these cells infinitely produce both rapidly proliferating tumor cells and CSCs via asymmetric division. CSCs are the major cause of drug resistance and tumor relapses [225]. Dormant CSCs can resist the cytotoxic effects of chemotherapy drugs because most chemotherapeutics target the cell proliferation machinery [226]. CSCs activate the multifunctional efflux transporter, adenosine triphosphate binding cassette (ABC) transporter [227]. Hence, CSCs can survive anticancer drug treatments and promote cancer recurrence [228]. Similar to pluripotent stem cells, CSCs express surface markers, including CD133, CXCR4, and CD44 [229,230,231]. CSC surface markers interact with external metabolites and the ECM, which activates oncogenic signaling pathways such as Wnt/β-catenin and promotes invasion and metastasis [232]. YAP/TAZ hyperactivation induces SOX9 expression to promote CSC properties [233]. Hence, CSCs have unique metabolic features that support stemness phenotypes.

Due to high metabolic plasticity, CSCs have diverse metabolic phenotypes determined by surface markers and tumor microenvironments [230]. The metabolic dependency of CSCs depends on cancer types and nutrient availability. Glycolysis is the major metabolic pathway used in CSCs. CSCs upregulate glucose uptake, lactate secretion, and glycolytic ATP production via CD44 surface marker [234,235]. Breast CSCs increase glycolytic enzyme expression, including pyruvate kinase isozymes M2 (PKM2) and LDHA [236]. Glioblastoma CSCs increase glycolytic enzymes via HIF-1 hyperactivation but decrease OXPHOS via mitochondria complex II inhibition [237]. However, some subtypes of leukemia, ovarian cancer, and pancreatic ductal adenocarcinoma-related CSCs have strong dependency toward OXPHOS [238]. The OXPHOS dependency of CSCs is supported by hyperactive BCL-2 and PGC-1α, which upregulate OXPHOS and mitochondrial biogenesis, respectively [239,240]. These metabolic dependencies can easily be switched. Thus, inhibiting glycolysis and OXPHOS simultaneously is important for targeting the metabolic plasticity of CSCs [241]. Leukemia stem cells are dependent on FAO via the fatty acid transporter, CD36 [242]. Through FAO, these cells can generate TCA cycle intermediates and mitochondrial membrane potential for ATP production. CD36-mediated FAO confers drug resistance to leukemia [243]. Thus, exploiting the CSC-specific metabolism that contributes to drug resistance, recurrence, and metastasis will provide effective anti-cancer therapies.

### 4.4. Drug Resistance

Common cancer therapeutic regimens include chemotherapy as a first-line treatment and targeted therapy as a secondary treatment. However, drug resistance inevitably arises as a critical obstacle [244]. Developing next-generation drugs for patients with drug-resistant tumors requires extensive resources. However, recent findings suggest that altering the metabolism can affect anticancer drug sensitivity. This treatment approach could bypass metabolic reprogramming that allows cancer cells to avoid the cytotoxicity of anticancer drugs and synergistically overcome metabolic plasticity and drug resistance [245].

Chemotherapeutics and targeted therapies induce cellular stress and apoptosis via various mechanisms. Cisplatin is a widely used chemotherapy drug that binds to glutathione or DNA and induces oxidative stress and DNA damage. Cisplatin is prescribed for lung, breast, and ovarian cancers [246]. However, in many cases, cancers adapt to the cytotoxic effects of cisplatin by upregulating cellular redox homeostasis [247]. In cisplatin-resistance cells, NADPH generation via the PPP suppresses the cytotoxic effects of cisplatin [248]. The uptake of cysteine is elevated by the activation of xCT antiporter, which is the rate-limiting precursor for glutathione synthesis [247]. Hence, inhibiting glutathione synthesis sensitizes the cytotoxicity of cisplatin [249]. In breast cancer, the RTK inhibitor lapatinib is used for targeted therapy [250]. Similar to almost all targeted therapeutic drugs, lapatinib therapy also results in drug resistance [251]. Increased glycolysis is a common characteristic of lapatinib resistance. Expression of glycolytic enzymes (e.g., GLUTs and LDHA) are upregulated in lapatinib-resistant breast cancer [252]. Lapatinib degrades ERRα in the nucleus. However, in resistant cells, hyperactivated ERRα is stabilized and activated via mTOR pathway. ERRα upregulates glutamine metabolism to supply TCA cycle intermediates and activate OXPHOS. Lapatinib-resistant cells activate their antioxidant metabolic pathways (e.g., NADPH synthesis) to avoid oxidative stress generated by OXPHOS [253].

## 5. Therapeutic Interventions Targeting Cancer Metabolism

The importance of cancer metabolism is increasingly emphasized as an outcome of oncogenic signaling and in terms of its effects on cancerous phenotypes. Although some metabolic phenotypes of cancer (e.g., aerobic glycolysis) offer diagnostic opportunities, aberrant metabolism-mediated cancer progression and drug resistance highlight urgent needs for development of metabolism-targeting anticancer drugs.

Studies and ongoing clinical trials aim to identify novel metabolism-targeting drugs. Metabolic enzymes within the glucose, glutamine, and fatty acid metabolic pathways are considered attractive drug targets. Cancer cells upregulate glycolytic enzyme expression and their enzymatic activities to facilitate aerobic glycolysis. For example, 2-Deoxy-Glucose (2DG), which binds and inhibits HK2, decreases glycolysis, and eventually induces ROS-mediated apoptosis in multiple cancer types (e.g., prostate cancer) [254,255]. Regeneration of NAD^+^ is crucial for aerobic glycolysis. LDH catalyzes the pyruvate to lactate conversion and regenerates NAD^+^. Drugs to inhibit LDH enzymatic activity are being examined. PSTMB induces apoptosis of lung cancer, breast cancer, colon cancer, and melanoma cells via LDH inhibition [256]. FX-11, galloflavin, and gossypol have anticancer effects via LDH inhibition [257,258]. Lactate, which is the final product of aerobic glycolysis, is secreted into the tumor microenvironment via MCT-1 transporter. When combined with metformin, MCT-1 inhibitor AZD3965 blocks lactate-mediated tumor progression and has significant anticancer effects [259,260]. Glutamine metabolism is also important for unrestrained cancer growth. Glutamine metabolism participates in various cellular processes, including the TCA cycle, fatty acid synthesis, and redox homeostasis. Blocking nutrient uptake is a powerful strategy for inhibiting specific metabolic pathways. L-g-Glutamyl-p-nitroanilide (GPNA) and V-9302 were developed for the inhibition of the glutamine transporter SLC1A5 and have anticancer effects in various cancer cell lines [261]. In the mitochondria, glutamine is transformed into glutamate by GLS and other transaminase enzymes. CB-839 and BPTES inhibit GLS and exhibits anticancer effects in triple negative breast cancer [262]. Aminooxyacetic acid (AOA) inhibits transaminase activity and thereby downregulates amino acid and nucleotide biosynthesis [88]. Recent results indicate the importance of de novo fatty acid synthesis for cancer progression. FASN mediates the elongation step of fatty acid synthesis and is a potential druggable target. C75 and orlistat are used for FASN inhibition; the FASN inhibitor, FASNall, effectively reduces tumor burdens in HER2^+^ breast cancer [263,264,265]. The cholesterol-synthesizing mevalonate pathway is highly deregulated in various cancer types. Statins, which were originally used as cholesterol-lowering medications, inhibit cancer growth by downregulating HMG-CoA reductase, the rate-limiting enzyme of the mevalonate pathway [266]. Part of statins’ anticancer effects result from the indirect suppression of the Hippo pathway. Mevalonate pathway inhibition reduces the lipidation of RhoA GTPase and thereby inhibits the transcriptional coactivator YAP/TAZ [121].

In contrast to Warburg’s early theory, studies find that OXPHOS is intact in cancer cells and that some types of cancers rather rely on OXPHOS for bioenergetics. Therefore, mitochondrial OXPHOS inhibition is a possible target for drug development. Oligomycin and biguanides inhibit the mitochondrial electron transport chain via inhibition of complex V and complex I, respectively, and thereby reduce mitochondrial ATP production [267]. OXPHOS inhibitors have anticancer effects in various cancer cells. OXPHOS inhibition-mediated energy stress induces AMPK activation. Given that AMPK inhibits cellular anabolic metabolism, including fatty acid synthesis, the mevalonate pathway, and GLUT1 expression, these OXPHOS inhibitors can simultaneously suppress multiple metabolic pathways [268].

Metabolic plasticity can compensate for the metabolic restrictions by upregulating other metabolic pathways to avoid stress responses. For example, metformin, a type 2 diabetes drug, suppresses OXPHOS by inhibiting mitochondria complex I, but glycolysis increases as a compensatory mechanism. Study findings suggest that combination therapy can be used to overcome metabolic plasticity. These therapies use combinations of metabolic pathway inhibitors to prevent metabolic compensation [269]. Glutamine metabolism is upregulated in Myc-induced liver cancer. GLS inhibition remarkably increases tumor free survival but upregulates compensatory mechanisms such as transamidases, HK2, FASN, and serine/glycine synthesis. Simultaneous inhibition of these pathways using combination therapy could synergistically suppress tumor growth [270].

Dietary interventions are also under investigation. Cancer cells are highly influenced by the nutrient status of the surrounding environment. Because food uptake regulates nutrient concentration in the blood, diet composition is a major factor that affects metabolite concentrations within tumor microenvironments [271]. Fasting synergistically increases the cytotoxic effects of chemotherapeutic drugs, including doxorubicin [272]. Fasting mimicking diet (FMD) and intermittent fasting also sensitize anticancer medicines and have significant anticancer effects. FMD reduces HO-1, which promotes cancer metastasis by disturbing redox homeostasis, and activates tumor-infiltrating lymphocytes [273]. Similarly, intermittent fasting inhibits insulin and IGF-1 signaling [274]. Consistent with these results, studies find synergistic effects between dietary interventions and metabolic drugs. For example, the combination of metformin and intermittent fasting is effective at targeting the metabolic plasticity of cancer. This combined therapy induces cytotoxic effects via the MCL1-apoptosis axis [269]. Therefore, elucidating altered drug efficacy under a differential metabolic context will be important not only for future drug development and preclinical studies, but also the repositioning of previous FDA-approved drugs.

## 6. Conclusions

In this review, we discussed aberrant metabolic phenotypes of cancer, underlying mechanisms, and therapeutic modalities. Abnormal metabolic activities are caused by deregulated oncogenic/tumor suppressor signaling pathways, including Hippo, Myc, PI3K/AKT, p53, and AMPK/LKB1. Metabolic defects lead to tumor malignancy, metastasis, and drug resistance by supplying energy, building blocks, and redox potentials. However, because metabolic pathways consist of complex and sophisticated networks, targeting cancer metabolism to develop treatment strategies requires further intensive research. Cancer metabolism is based on differential metabolic stress responses between cancer cells and normal cells. Therefore, targeting cancer metabolism is expected to selectively inhibit cancer progression, with less cytotoxicity to normal cells. Metabolism-targeting drugs can induce cancer cell-specific cytotoxic effects. Cancer metabolism provides opportunities for next-generation anticancer therapies that could be further improved using combination approaches that simultaneously inhibit multiple metabolic pathways. Dietary intervention can be used as an adjuvant treatment with traditional chemotherapy and targeted therapies. Given their high heterogeneity, cancer cells have diverse genetic mutations, even within the same tumor mass. Metabolic phenotypes of cancer are significantly affected by patient-specific tumor microenvironments. The metabolic status of one patient can be very different from that of another patient. Establishing a metabolism-based precision medicine platform that can best predict personalized drugs, which synergize with a patient’s metabolic status, should be the goal of future research.

## Figures and Tables

**Table 1:** Interaction between signal transduction pathway and metabolic enzymes.

#	Signal Transduction	Target Metabolic Enzymes	Phenotype
1	YAP/TAZ	GLUT3 expression [63]	Increasing glucose uptake
		HK2 expression [64]	Increasing glycolysis
		PFKFB3 expression [65]	Increasing glycolysis
		SLC1A5, SLC7A5 expression [66,67]	Increasing glutamine uptake
		GOT1, PSAT1 expression [68,69]	Producing NEAA and TCA cycle intermediates
		Bile acid production [70]	Increasing metastatic potential
2	PI3K-AKT	mTORC1 activation [71]	Increasing protein translation
		GSK3 inhibition [72]	Inhibiting glycogen synthesis
		AS160 activation [73,74,75]	Increasing membrane trafficking of GLUT
		TXNIP inhibition [76]	Enhancing GLUT1/4 membrane localization
		HK2 and PFKFB activation [16,17]	Increasing glycolysis
		HIF-1α activation [77]	Increasing glycolytic enzyme expression
		Myc expression and stabilization [78,79,80]	Increasing glutamine metabolism
		ACLY activation [81]	Increasing de novo lipid synthesis and histone acetylation
		SREBP stabilization [82,83]	Increasing fatty acid metabolism
3	Myc	GLUT, HK2, PFK expression [11,84]	Increasing glycolysis
		LDH and MCT1 expression [85,86]	NAD^+^ regeneration
		G6PD and TKT [87]	Increasing PPP efficiency
		OGT [24,27]	Increasing glycosylation
		SLC1A5 and SLC38A5 expression [5]	Increasing glutamine uptake
		GLS expression [43]	Increasing glutaminolysis
		GLUD and transaminase [34,88]	Producing NEAA and TCA cycle intermediates
4	p53	Inhibiting GLUT1/4 expression [89]	Inhibiting glucose uptake
		TIGAR expression [90]	Inhibiting PFK and glycolysis
		Inhibits LDH and PDH [91]	Inhibiting TCA cycle influx
		CPT1 and LPIN1 expression [6,92]	Increasing Fatty acid oxidation
		SCO2 expression [93]	Increasing OXPHOS and accumulates NADH and FADH2
5	LKB1-AMPK	ACC inhibition [94]	Inhibiting fatty acid synthesis and ATP consumption
		CD36 [95,96]	Increasing fatty acid uptake
